# A Prediction Model for Membrane Proteins Using Moments Based Features

**DOI:** 10.1155/2016/8370132

**Published:** 2016-02-15

**Authors:** Ahmad Hassan Butt, Sher Afzal Khan, Hamza Jamil, Nouman Rasool, Yaser Daanial Khan

**Affiliations:** ^1^Department of Computer Science, School of Systems and Technology, University of Management and Technology, P.O. Box 10033, C-II, Johar Town, Lahore 54770, Pakistan; ^2^Faculty of Computing and Information Technology in Rabigh, King Abdul Aziz University, Saudi Arabia; ^3^Department of Chemistry, School of Science, University of Management and Technology, P.O. Box 10033, C-II, Johar Town, Lahore 54770, Pakistan

## Abstract

The most expedient unit of the human body is its cell. Encapsulated within the cell are many infinitesimal entities and molecules which are protected by a cell membrane. The proteins that are associated with this lipid based bilayer cell membrane are known as membrane proteins and are considered to play a significant role. These membrane proteins exhibit their effect in cellular activities inside and outside of the cell. According to the scientists in pharmaceutical organizations, these membrane proteins perform key task in drug interactions. In this study, a technique is presented that is based on various computationally intelligent methods used for the prediction of membrane protein without the experimental use of mass spectrometry. Statistical moments were used to extract features and furthermore a Multilayer Neural Network was trained using backpropagation for the prediction of membrane proteins. Results show that the proposed technique performs better than existing methodologies.

## 1. Introduction

Among different macromolecules which constitute the cell, proteins are structural and functional unit of the cell. Proteins carry out thousands of chemical reactions and process both inside and outside the cell. Each cell is enclosed by a protective wall that consists of lipids and is named as plasma membrane. Nevertheless, very few lipid soluble and nonpolar molecules can get entry by direct diffusion through lipid bilayer. Most of the time, this transferring of molecules through membranes is performed by membrane proteins [1]. Approximately 25–75% of the mass of the membrane consists of proteins. These proteins may be integral or peripheral. These proteins may act as receptor and play important role in cell signaling. Glycoproteins are responsible for cell-cell adhesion. Some proteins play important role in transportation of molecules across the cell membranes. These proteins may be carriers, channels, or pumps. About 60–70% of these proteins consist of *α* helices; very few consist of *β* barrels [2].

Amino acids are the structural component of each protein. The structure of protein is strictly linked with the function of that protein. If the nature or sequence of protein is changed, the function of protein may alter. There are four types of structural organizations in proteins which are primary, secondary, tertiary, and quaternary level of structures [1]. The protein sequence specifies the particular function and the shape of the protein structure. Informally, proteins can be categorized into three different classes: globular, fibrous, and membrane proteins. These protein types are related with each other but have discriminations in their tertiary structures [3–5]. Globular proteins are mostly enzymes. Fibrous proteins are often structural and are mostly part of some cellular structure. Membrane proteins are responsible for signaling between the cells and act as channels for polar and nonpolar molecules to be transported through the cell membrane [6, 7]. Particularly, the advancements in molecular biology have led to the rapid increase of protein sequences in databanks. These protein sequences are used to extract various features related to that protein. The total protein sequences in Swiss-Prot databank were 3,939 in 1986. In accordance with the version 2015_03 released on March 4, 2015, the number of total protein sequences has reached 547,964 sequence entries.

The prediction of membrane protein is a complex problem and therefore needs the prediction model to be accurate and efficient. Most of cellular functionalities are performed through these significant membrane proteins. The main target of many pharmaceutical research organizations is membrane protein because 50% of the drugs have their targets as membrane proteins [8–10]. Every membrane protein type has its specific behavior and functionality with the cell membrane. Many systems have been proposed in [11–14] to determine the exact purpose and behavior of the membrane protein within the membrane and outside the membrane and have produced results with accuracy but there are still many areas where this accuracy can be improved and efficient results could be achieved.

The proposed system endeavors to predict whether a given protein sequence corresponds to a membrane or a nonmembrane protein producing assiduous and efficient results as compared to existing systems. Firstly, various sequence specific and content specific features are extracted like the Position-Relative-Incident-Matrix (PRIM) and Frequency-Matrix (FM) from the protein input query. After successful feature extraction, these feature vectors are clamped to a neural network for comprehensive training and subsequent classification of arbitrary protein sequence received as input.

In many previous efforts, membrane proteins are predicted through their primary sequence of amino acids. Most of these techniques were based on the compositions of* Amino Acids* (AAs) [15] and* Pseudo-Amino-Acid* (PseAA) [16]. Chou and Elrod in [15] proposed the method of membrane protein type prediction based on amino acid (AA) compositions. This work is considered to be a pioneer for protein prediction methods based on amino acids composition. They used Covariant-Discriminant-Analysis (CDA) in combination with AA composition which represented the frequencies of the incidence of AAs in the primary sequence. The problem with this method was the loss of information regarding the protein sequence which directly affected the efficiency of the prediction model. To preserve the order of sequence and its information, Chou proposed PseAA composition in [16] which had an impact on enhancement of prediction of the protein sequence information. Chou implemented Augmented CDA, least Hamming distance, least Euclidean distance [17], and ProtLock [18] with PseAAC through which improvement in the outcomes was examined. Cai et al. in [19] used the PseAA and Functional Domain (FD) compositions with Support Vector Machine (SVM) for membrane protein type prediction. In [20] Cai et al. applied Support Vector Machines with AA composition features. Wang et al. in [21] used variation of SVM with weights for identifying membrane proteins using PseAA composition features. Supervised Locally Linear Embedding (SLLE) with Nearest-Neighbor classifier was used by Wang et al. in [22] for feature extraction and classification of membrane proteins. Chou and Cai in [23] used patterns based on amphipathic effects of sequence orders to alleviate their existing methodologies. These amphiphilic effects were used with PseAA composition which restrain information related to the hydrophobic and hydrophilic associative features and notably increased the prediction process of membrane protein types. Liu et al. in [24, 25] introduced Fourier spectrum and low-frequency Fourier spectrum analysis based on the PseAA compositions. The major benefit of this analysis was to utilize the pattern information of protein sequence more efficiently. Chou and Cai in [26] proposed a hybrid method for predicting membrane proteins using GO-PseAA which was proposed and used in [27–29] based on combination of PseAA composition and Gene-Ontology (GO). This method proved to be better in accuracy for identifying the five membrane protein types. Shen and Chou in [30] introduced Optimized Evidence-Theoretic *K*-Nearest-Neighbor (OET-*K*NN) classifier using PseAA compositions which was based on the evidence theory. Shen et al. in [31] applied fuzzy *K*-Nearest-Neighbors (*K*NN) algorithm combined with PseAA compositions. This technique was based on fuzzy mathematics and yielded an improved approach in process of the membrane protein type prediction. In [32] Wang et al. proposed a novel approach called “Stacked generalization.” This method used combinations of several classifiers as a meta-classifier in order to increase the performance of generalization. Yang et al. in [33] used AA and dipeptide composition based feature for their membrane protein prediction methods. Pu et al. in [34] used Integrated Approach for Membrane Protein Classification (IAMPC). They used Position-Specific-Scoring-Matrix (PSSM) based on the protein AA sequences which proved to be a better approach than Functional Domain feature extractions. Chou and Shen in [35] implemented a Web server (MemType-2L) which was used as a two-layer predictive engine. The first phase was used to identify the protein sequence as a membrane protein or nonmembrane protein and second phase differentiated the specific membrane protein type. The server was based on the features extracted through Pse-PSSM (Pseudo-Position-Specific-Scoring-Matrix) with combination of an ensemble classifier.

## 2. Material and Methods

The benchmark dataset used in proposed system for training and testing was created by Chou and Shen in [35]. The protein sequences were collected from version 51.0 released on October 6, 2006, of Swiss-Prot database. The following criteria were used to collect high-quality data and much desired information working dataset. In the first phase, the sequences with annotation like “fragment” were not included. Proteins with less than 50 amino acid residues sequences were also not included. In second phase, such sequences that were annotated with ambiguous terms like “potential”, “probable”, “probably”, “may be”, or “by similarity” were not considered as a part of this dataset. The sequences screened after the above procedures were kept in membrane proteins dataset if they were annotated with term “membrane protein” and the rest of the sequences that were not annotated with this term were stored as dataset for nonmembrane proteins. In order to remove the homology and redundancy bias, reduction sequences which have 80% identity in sequence with any other membrane proteins were left out. Similar procedure was followed in the nonmembrane dataset to remove redundant sequences. Finally, the dataset containing 15,547 protein sequences was built in which 7,582 were membrane proteins and 7,965 were nonmembrane proteins. This dataset is the latest benchmark dataset and is currently being used in mostly predictive systems built to predict membrane proteins. Chou and Shen also prepared an independent test dataset of 4,333 membrane proteins.

### 2.1. Feature Extraction

The following feature extraction methodologies are used to determine features or patterns linked with any specific protein. These methods are discussed below.

#### 2.1.1. Statistical Moments

Many proposed techniques in pattern recognition consider that statistical moments are useful to generate features from a given pattern which are not dependent upon any parameters. Many researchers have used moments to capture important features and characterize the functionalities of any given pattern [36, 37]. Moments are certain types of biased average that are used for the analysis of the concentrations of some major configurations in pattern recognition related problems. For various pattern recognition systems and object representations, orthogonal moments are considered as a valuable technique. In recent study, it has been observed that discrete orthogonal moments have produced better results than the continuous orthogonal moments for discrete and quantized data. These discrete orthogonal moments have the ability to transform the object representations with minimum amount of loss of information [38].

In order to compute two-dimensional moments, the one-dimensional primary structure is translated into a two-dimensional structure using a row major scheme. The dimension of the two-dimensional matrix is computed by taking the square root of the length of protein

(1) $$\begin{array}{c} n=\left\lceil \;\sqrt{k}\;\right\rceil , \end{array}$$

where *n* is the dimension of the two-dimensional square matrix and *k* is the length of the polypeptide chain.

There are many different forms of moments that can be calculated through any matrix or collection of vectors that represent any pattern. The most common of the moments are raw moments which are computed from the following:

(2) $$\begin{array}{c} M_{xy}=\underset{i}{\sum }\underset{j}{\sum }i^{x}j^{y}f\left(\;i,j\;\right). \end{array}$$

The raw moments assume the origin of data as the reference point while the distance components from the origin are used to compute moments. The central moments use the centroid of the data as the reference point and are computed from the following equation:

(3) $$\begin{array}{c} \mu_{xy}=\underset{p}{\sum }\underset{q}{\sum }{\left(\;p-\overset{-}{p}\;\right)}^{x}{\left(\;q-\overset{-}{q}\;\right)}^{y}f\left(\;p,q\;\right). \end{array}$$

Here $\overset{-}{p}$ and $\overset{-}{q}$ form the centroid and are calculated from

(4) p−=M10M00,q−=M01M00.

The one-dimensional notation was transformed into a square matrix notation so that Hahn moments could be computed. Two-dimensional Hahn moments are orthogonal moments that require a square matrix as two-dimensional input data. The Hahn polynomial of order *n* is given as

(5) hnμ,νr,N=N+υ−1nN−1n·∑k=0n−1k−nk−rk2N+μ+υ−n−1kN+υ−1kN−1k1k!.

The above expression uses the Pochhammer symbol generalized as

(6) $$\begin{array}{c} {\left(\;a\;\right)}_{k}=a\left(\;a+1\;\right)\cdots \left(\;a+k-1\;\right). \end{array}$$

And it is simplified using the Gamma operator

(7) $$\begin{array}{c} {\left(\;a\;\right)}_{k}=\frac{\Gamma \left(a+k\right)}{\Gamma \left(a\right)}. \end{array}$$

The raw values of Hahn moments are usually scaled using a weighting function and square norm given as

(8) $$\begin{array}{c} \tilde{h_{n}^{\mu ,\nu }}\left(\;r,N\;\right)=h_{n}^{\mu ,\nu }\left(\;r,N\;\right)\sqrt{\frac{\rho \left(r\right)}{d_{n}^{2}}},\;n=0,1,\ldots ,N-1. \end{array}$$

Meanwhile,

(9) $$\begin{array}{c} \rho \left(\;r\;\right)=\frac{\Gamma \left(\mu +r+\upsilon \right)\Gamma \left(\upsilon +r+1\right){\left(\mu +\upsilon +r+1\right)}_{N}}{\left(\mu +\upsilon +2r+1\right)n!\left(N-r-1\right)!}. \end{array}$$

The orthogonal normalized Hahn moments for the two-dimensional discrete data are computed using the following equation:

(10) Hij=∑q=0N−1 ∑p=0N−1βpqhiμ,ν~q,Nhjμ,ν~p,N,m,n=0,1,…,N−1.

The central moments and the Hahn moments are computed up to order 3.

#### 2.1.2. Position-Relative-Incident-Matrix (PRIM) and Frequency-Matrix (FM)

The first step in extraction of features is to compute the matrix formation of the input protein query. For this purpose, the length of the protein sequence is used to build the PRIM and FM. These matrices are then used for the calculation of moments through which feature vectors are formed. A protein sequence *S* with total *N* amino acid residues is represented through PRIM as follows:

(11) SPRIM=A1→1A1→2⋯A1→j⋯A1→20A2→1A2→2⋯A2→j⋯A2→20⋮⋮⋯⋮⋯⋮Ai→1Ai→2⋯Ai→j⋯Ai→20⋮⋮⋯⋮⋯⋮AN→1AN→2⋯AN→j⋯AN→20.

In the given protein sequence, the indication of the score of the *i*th position residue is determined by *A*_*i*→*j*_. In the biological evolutionary process, this score is substituted by amino acid type *j*. The values of *j* = 1,2,…, 20 are the representation of the alphabetical order of 20 native amino acids.

After the feature vector is obtained, it is trained and classified through Multilayered Neural Network (MLNN) for membrane and nonmembrane protein predictions.

#### 2.1.3. Neural Networks with Backpropagation (BP)

**Figure 1:**
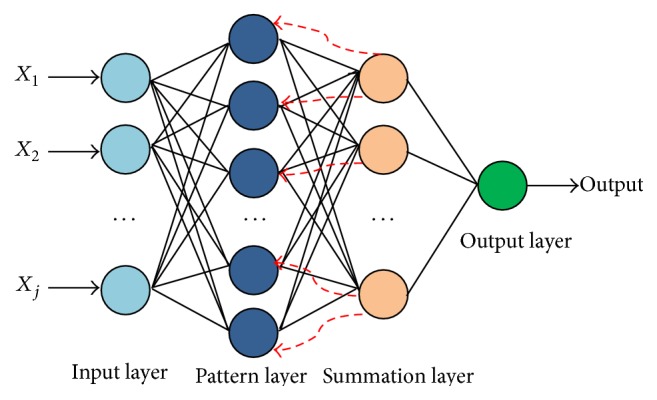
The four-layer architecture of MLP with backpropagation.

In classifications of pattern recognition problems, neural networks are amongst the mostly used methodologies. These neural network systems are nonlinear-adaptive and are capable of approximating any function. The BP training algorithm is very well known for Multilayer Feed-Forward Neural Networks and was introduced by [39]. The backpropagation neural network (BPNN) and Feed-Forward Neural Network (FFNN) are similar which contain an input layer, multiple hidden layers, and the output layer as shown in Figure 1. The hidden layers consist of selected number of neurons. These neurons act as the core processing elements of the network. These neurons or nodes form a constellation through connectivity in between the layers. Through the incoming connections of the node, it receives the weighted activations of the previous layer nodes. These weights are summed up and the result is passed on through an activation function. The outcome of this process is the activation of the node. The specific weight is then multiplied with this activation value for every connection, which is outgoing, and is then transferred to the next node. For a MLNN, the activation or threshold function that is used should be nonlinear. If the threshold function is not nonlinear, then the MLNN will perform as a single layer network. The most commonly applied threshold or activation function is the logistic function sigmoid which is defined by the following formula:

(12) $$\begin{array}{c} \sigma \left(\;n\;\right)=\frac{1}{1+e^{-n}}, \end{array}$$

where *n* is the number of given inputs to the network. There are many threshold functions available but this is the most commonly used and has been very useful in BPNN learning. During the BP training, every pattern is trained one at a time. An epoch is the training of all the input patterns to a network of the training dataset. The BPNN uses gradient decent algorithm. It uses various attempts to reduce its error along its gradient in order to improve the overall performance of the neural network. This error can be expressed as root-mean-squared-error (RMSE) which is formulated as

(13) $$\begin{array}{c} E=\frac{1}{2}\underset{p}{\sum }{\left\|\;t_{p}-o_{p}\;\right\|}^{2}. \end{array}$$

Here *E* is sum of errors of half of the averages of the projected target (*t*) and output vector (*o*) difference of all patterns (*p*). In the start of the BP training, the weights are set randomly. The weights (*w*) are altered towards the maximum decrease direction and are scaled by the adaptive learning rate lambda (*λ* = 0.01):

(14) ∇E=δEδw1,δEδw2,…,δEδwn,wnew=wold−λ∇E.

The derivative property of sigmoid function is

(15) $$\begin{array}{c} \frac{d}{dn}\sigma \left(\;n\;\right)=\sigma \left(\;n\;\right)\left(\;1-\sigma \left(\;n\;\right)\;\right). \end{array}$$

By using the above method, the computation of the derivative of the logistic sigmoid function is performed. This method eases the efforts of computations in BP method. Hence, the weight change equations are reduced to

(16) ∇wfrom,to=−λofromδto,δoutput=−toutput−ooutput,δhidden=σ′shidden∑iδiwhidden,i.

Before the threshold function is applied to the unprocessed sum for each neuron, the value of this sum is stored. After storing these sum (*s*) values, the weight changes are performed sufficiently using the basic algebraic operations. The BP algorithm is reliable but not that fast in training. However, the various parameters can be changed in order to improve the speed of the overall training process.

## 3. Results and Discussions

In order to measure the error rates in proposed classification model for the performance evaluation, the data is divided into partitions. The most widespread statistical techniques are used to create partitions. Cross-validation is the partitioning technique that is commonly used in verifying the classification performance of a classifier. It is further used for testing with mutually exclusive folds partitioning in the dataset. To assess the performance of a developed model, there is a need of some method that will examine the prediction model to verify how well it performed. There are several parameters on which the assessment and estimates of the performance of classifiers are measured. The type of data and the classification signify the detail of which parameters to be used. To measure the performance of a classifier, the typically used tests and tools are Jackknife test, confusion matrix, and ROC curves.

### 3.1. Jackknife Test

Jackknife validation test is also known as leave-one-out cross test. In this test, data is divided into *n*-folds. The testing dataset instance is left out and the rest of the dataset instances are trained by the classification model. This whole process is performed *n* times. After the results of all the *n* predictions are acquired, they are further averaged for error identifications in estimates. Jackknife is the most severe and operational test in cross validation tests. Unique results are achieved through this test. The only drawback in considering Jackknife test for evaluation is the effectiveness of the computational cost of the testing process as *n* iterations are performed. This test was performed in order to verify the classifier performance. The results are mentioned in Table 1.

### 3.2. Accuracy

**Figure 2:**
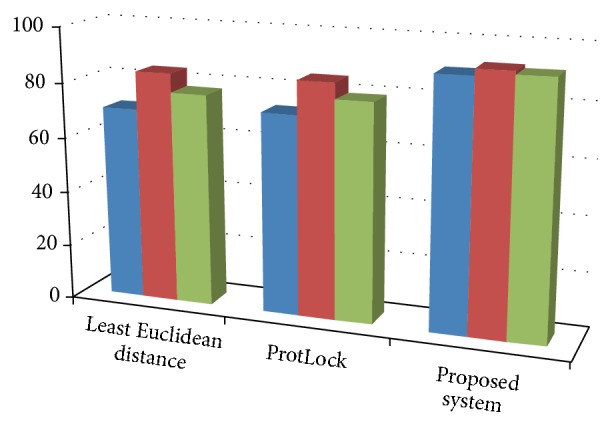
Histogram of 3D chart showing the accuracy of proposed system.

The accuracy or the error rate of a classifier is used to measure its performance. The degree of true predicted class of a classifier is measured by the accuracy of the system. It is the proportionality of predictions that are true in comparison with false ones. The accuracies computed after successful classifications are also mentioned in Table 1 whereas the histogram chart for accuracies is shown in Figure 2. It is formulated as below:

(17) $$\begin{array}{c} \text{Accuracy}=\frac{\text{TP}+\text{TN}}{\text{TP}+\text{TN}+\text{FP}+\text{FN}}\times 100. \end{array}$$

### 3.3. Confusion Matrix

Confusion matrix is mostly considered as a measuring tool for performance assessments of various classification algorithms. The classification outcomes are compared with the actual outcomes in confusion matrix. A matrix is used to represent this assessment where actual class is represented by each column while each row is the predicted class.

Table 2 shows the contingency table or the confusion matrix which includes True Positives (TP) which are true outcomes predicted as positive outcomes and True Negatives (TN) which are false outcomes predicted as negatives. False Positive (FP) outcomes are false but are predicted as positive outcome which is an error. Such types of errors are known as Type I errors. Also, False Negative (FN) outcomes are those outcomes which are true but are predicted as negative outcomes. This is also an error which is known as Type II error. The confusion matrix in Table 3 was obtained after the successful classification of membrane proteins from nonmembrane proteins.

### 3.4. ROC (Receiver-Operating-Characteristic) Curves

**Figure 3:**
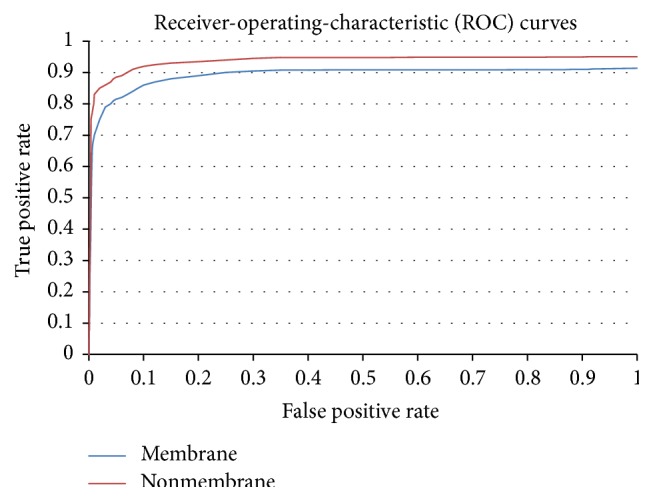
ROC curves of the neural networks in membrane protein classification.

In order to review the core gears of classification methods, a diligent tool was used, namely, the ROC curve, to highlight the accuracy of the system. The accuracy of the classifier can be envisioned in a broad way by using this tool. The ROC curve uses a plot of sensitivity (true positive rate) on the vertical axis and false positive rate on the horizontal axis of the plot. The classifier performance is considered accurate at best when the curve area is a bit closer to the left top corner. The ROC curves are shown in Figure 3 which are obtained after performing classifications.

## 4. Conclusions and Future Work

In this research work, the proposed systems yield better results for the problem of membrane protein prediction. The importance of such prediction systems is useful in the identification of protein functionality which is valuable in process of drug discovery. In this system, various computational techniques have been used and are curtailed within the field of pattern recognition. The results produced in currently proposed system are more accurate and robust as compared to previous results from [17, 18] in Table 1. As the biological sequence data is growing at enormous pace in various databases like Swiss-Prot databank, the room for efficiency and possibilities for improvements in this area still exist in the coming future. In facilitation of scientist community for their experimental purposes and the student community for their research objectives, we shall develop user-friendly webserver introducing our novel method of prediction presented in this paper.

## Figures and Tables

**Table 1:** Comparison in terms of accuracy with existing systems on benchmark dataset.

Protein	Least Euclidean distance [17] (%)	ProtLock [18] (%)	Proposed system (%)
Membrane	70.2	72.7	90.0
Nonmembrane	84.0	84.8	92.4
Overall	77.2	78.9	91.23

**Table 2:** Contingency table or matrix of confusion.

	Predicted class		
	Total outcomes	Condition, positive	Condition, negative
Actual class	Test outcome, positive	TP	FN (error type I)
	Test outcome, negative	FP (error type II)	TN

**Table 3:** Confusion matrices of the neural networks in membrane protein classification.

	Target class			
	Total outcome	Condition, positive	Condition, negative	Total percentage
Output class	Test outcome, positive	**6824**	**605**	**91.85%**
		43.89%	3.89%	**8.15%**
	Test outcome, negative	**758**	**7360**	**90.66%**
		4.87%	47.34%	**9.34%**
	Output accuracy	**90.0%**	**92.4%**	**91.23%**
		**10.0%**	**7.6%**	**8.77%**
